# Admixed phenotype of *NEDD4L* associated periventricular nodular heterotopia: A case report

**DOI:** 10.1097/MD.0000000000026136

**Published:** 2021-06-04

**Authors:** Martina Pecimonova, Jan Radvanszky, David Smolak, Jaroslav Budis, Michal Lichvar, Diana Kristinova, Ivica Rozova, Jan Turna, Tomas Szemes

**Affiliations:** a Geneton Ltd.; b Department of Molecular Biology, Faculty of Natural Sciences, Comenius University; c Institute of Clinical and Translational Research, Biomedical Research Center, Slovak Academy of Sciences; d Comenius University Science Park; e Slovak Centre of Scientific and Technical Information; f Medirex a.s., Bratislava, Slovakia.

**Keywords:** E3 ubiquitin-protein ligase NEDD4-like, *NEDD4L*, periventricular nodular heterotopia-7, PVNH7

## Abstract

**Rationale:**

Periventricular nodular heterotopia-7 (PVNH7) is a neurodevelopmental disorder associated with improper neuronal migration during neurogenesis in cortex development caused by pathogenic variants in the *NEDD4L* gene.

**Patient concerns:**

We report the case of a polystigmatized 2-year-old boy having significant symptomatologic overlap with PVNH7, such as delayed psychomotor and mental development, seizures and infantile spasms, periventricular nodular heterotopia, polymicrogyria, cleft palate, 2 to 3 toe syndactyly, hypotonia, microretrognathia, strabismus, and absent speech and walking. The patient showed also distinct symptoms falling outside PVNH7 symptomatology, also present in the proband's older brother, such as blue sclerae, hydronephrosis, transversal palmar crease (found also in their father), and bilateral *talipes equinovarus*. In addition, the patient suffered from many other symptoms.

**Diagnoses:**

The boy, his brother and their parents were subjected to whole-exome sequencing. Because of uncertainties in symptomatology and inheritance pattern, the top-down approach was hard to apply. Using the bottom-up approach, we identified a known pathogenic variant, NM_001144967.2(NEDD4L):c.2677G>A:p.Glu893Lys, in the proband's genome that absented in any other analyzed family member, suggesting its de novo origin.

**Interventions and outcomes:**

The patient was treated with Convulex 300 mg/mL for the successful seizure control and Euthyrox 25mg for the treatment of thyroid malfunction. He also took various supplements for the metabolism support and digestion regulation. Moreover, the patient underwent the corrective surgeries of cleft palate and *talipes equinovarus*.

**Lessons:**

We successfully identified the causative mutation NM_001144967.2(NEDD4L):c.2677G>A:p.Glu893Lys explaining symptoms overlapping those reported for PVNH7. Symptoms shared with the brother were not explained by this variant, since he was not a carrier of the pathogenic *NEDD4L* variant. These are most likely not extended phenotypes of PVNH7, rather an independent clinical entity caused by a yet unidentified genetic factor in the family, highlighting thus the importance of thorough evaluation of symptomatology and genomic findings in affected and unaffected family members, when such data are available.

## 1 Introduction

Periventricular nodular heterotopia-7 (PVNH7, OMIM#617201) is an ultra-rare neurodevelopmental disease affecting proper neuronal migration during neurogenesis in cortex development caused by impaired E3-ubiquitin ligase NEDD4L (Neural Precursor Cell Expressed, Developmentally Downregulated 4; UniProtKB - Q96PU5). This results in various abnormal phenotypic features that affect patients in varying degrees (overviewed in Supplementary Table S1), at least according to the few cases published in the literature. Broix et al described 5 unrelated patients and 2 siblings who developed PVNH7. All patients suffered from bilateral periventricular nodular heterotopia and developmental delay, 3 of them from a severe form. The patients presented predominantly with toe syndactyly, cleft palate, seizures and hypotonia. Individual patients developed various additional features including brain anomalies, optic and hearing impairments, dysmorphic features, arthrogryposis and cryptorchidism.^[1]^ Kato et al reported a case of a Japanese girl with severe developmental delay, periventricular nodular heterotopia, polymicrogyria, infantile spasms, cleft palate and *patent foramen ovale*. In addition, she presented with hypotonia, hypsarrhythmia, unstable neck and a difficulty maintaining eye contact.^[2]^ Elbracht et al described a female patient who suffered from severe developmental delay, bilateral periventricular nodular heterotopia, cleft palate and mild 2 to 3 toe syndactyly. Moreover, she had several morphological anomalies such as low posterior hairline, short neck, mild plagiocephaly, bilateral *talipes equinovarus*, sandal gap, adducted thumbs and truncal hypotonia. In addition, she presented with hypertonia on both legs and hyperactive tendon reflexes. By retrospective analysis signs of PVNH7 were also found in a fetus from the couple's previous pregnancy, which was terminated prematurely due to hypokinesia, upper limbs contractures, mild retrogenia and mildly thick *cavum septi pellucidi*.^[3]^ Recently, 6 more PVNH7 patients were reported. Ma et al. reported a patient suffering from global developmental delay, intellectual disability, cleft palate, seizures and hypotonia.^[4]^ Stouffs et al reported 5 patients of which 4 were related. All patients suffered from intellectual disability, periventricular nodular heterotopia and syndactyly, predominantly between 2nd and 3rd toes except a patient with 3 to 4 and 3 to 4 to 5 syndactyly. Three patients were diagnosed with convulsions and/or seizures. Two patients had prominent/high forehead and developed perisylvian polymicrogyria. Other features present in any patient includes macrocephaly, 5th finger clinodactyly, penoscrotal hypospadias with bifid chordee, hypotonia and absent speech.^[5]^

## 2 Case presentation

Herein we report a case of a polystigmatized 3 year-old boy, who was born by cesarean section in the 37th week of gestational age of a risk pregnancy, marked by oligohydramnios and growth retardation with a birth weight of 2680 g, length of 45 cm and Apgar score 8 of 10. During the first weeks of pregnancy the mother was taking oral hormonal contraceptive Dienorette (Laboratorios Leon Farma S.A., ESP), and reported also occasional alcohol and tobacco use. Following birth, the proband had numerous visible anomalies, such as thoracic left convex scoliosis, microretrognathia, cleft of both soft and hard palate, blue sclerae, bilateral clinodactyly of the 5th finger, single transverse palmar crease, bilateral *talipes equinovarus*, and simple incomplete syndactyly of the second and third toes. The *talipes equinovarus* and the cleft palate were surgically corrected at the age of 2 and 11 months, respectively. Other clinical features included horizontal nystagmus, hypermetropy, strabism, hypotonia with hypotonic quadriparesis and acral hypertonus, mild dyspnea, gastroesophageal reflux, hydronephrosis, and obstipated stool often with blood admixture. The patient was reported of having subclinical hypothyreosis, impaired cholesterol metabolism and hypoproteinemia with mildly decreased levels of total cholesterol, HDL cholesterol and apolipoproteins A and B. At the age of 2, speech and walking still absented, and patient showed signs of delayed psychomotor and mental development. At the age of 3, the proband was diagnosed with hypsarrhythmia and he developed epileptic seizures with eyelid myoclonia and infantile spasms. The patient was treated with Convulex 300 mg/mL for the successful seizure control and Euthyrox 25 mg for the treatment of thyroid malfunction. He also took various supplements for the metabolism support and digestion regulation.

**Figure 1:**
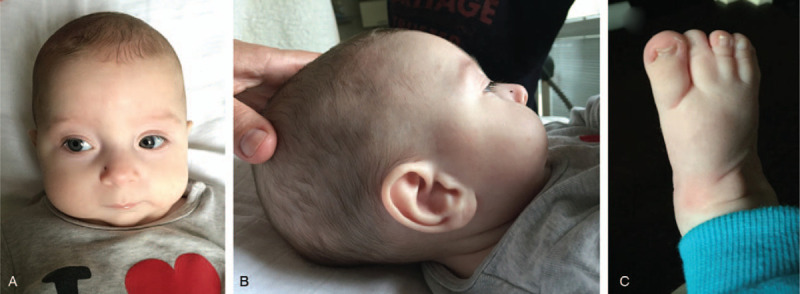
Some of the patient's external phenotypic features associated with PVNH7 around 5 months of age: (A) frontal and (B) lateral view of the head with visible microretrognathia; (C) 2nd and 3rd toe syndactyly.

Following the identification of the possible molecular cause of the symptoms (described below), the patient underwent further examinations such as electroencephalography (EEG) and magnetic resonance imaging (MRI) that confirmed the suspected diagnosis of PVNH7. Native and sleep EEG showed a severely pathological graph with encephalopathic character similar to Lennox-Gastaut syndrome. In the native EEG, slow basal activity in the delta range with poor regional differentiation and very frequent spike-wave complexes with slow frequency of 3 Hz and very high amplitude were observed, predominantly on the left side (mainly generalized). In the sleep EEG, transients were undetectable and epileptic activity was increased with no changes in the feature and localization. MRI confirmed the occurrence of supratentorial bilateral subependymal nodular to laminar lesions of cortex signal indicating the grey matter heterotopia with the width of 11 mm. In addition, numerous lesions showing nodular character located paraventricularly, in the central white matter and in *corona radiata* were observed. MRI revealed also thickened cortex bilaterally suggesting polymicrogyria without the signal changes, as well as hypoplasia of optic nerves and chiasma bilaterally. The unaffected parents reported no family history of known inherited diseases, abortions or developmental anomalies, except of the proband's older brother, who presented with hypotonia, blue sclerae, hydronephrosis, bilateral palmar crease (that was found also in their father), and bilateral *talipes equinovarus*. Supplementary Table S1 overviews the proband‘s complete symptomatology, together with the phenotype of his brother and the previously reported cases of PVNH7, while some of the external phenotypic features of the presently reported patient are visible on Figure 1.

Cytogenetic testing of the proband's genome revealed neither chromosomal aberrations nor numeric abnormalities. No pathogenic or likely pathogenic genomic imbalances on submicroscopic level were identified using array comparative genomic hybridisation. Whole-exome sequencing was then performed using DNA samples belonging to the proband, his brother and both his parents (details on the methodology can be found in Supplementary File 1). Conventional top-down strategy, that is, the phenotype and inheritance-pattern driven variant filtration and prioritization was complicated because of the then undiagnosed core feature of nodular heterotopia, slightly overlapping symptomatology of the brothers and the resulting ambiguity in inheritance-pattern. Therefore, we opted for the opposite approach, that is, the bottom-up strategy based on the identification and listing of all the proband's variants evidenced by ClinVar as pathogenic and likely pathogenic with subsequent manual searching for those consistent with proband's clinical manifestations, population data, and segregation patterns of the suspected variants and the partial phenotypes in the family. None of these analyses was performed due to research and all presented findings were results of the routine process of molecular diagnostics, therefore no ethics committee approval was needed.

In the patient's data set we identified a highly suspected heterozygous single nucleotide variant in the *NEDD4L* gene, NM_001144967.2(NEDD4L):c.2677G>A;p.Glu893Lys that was reported to be associated with PVNH7. This variant was not present in any other analysed family member suggesting a likely de novo origin, although we were not able to rule out genetic mosaicism in one of the parents. With regard to PVHN7, c.2677G>A (p.Glu893Lys) variant was classified either as “pathogenic” by 4 individual submissions in ClinVar database or as a “variant of uncertain significance” by single submitter (ClinVarID_225191). Human Gene Mutation Database classified it as a “disease causing mutation” (CM1613945), based on primary literature reports. Despite being present in dbSNP (rs879255597), the variant had no known frequency data at the time of the preparation of this manuscript (Build 154, Released on April 21, 2020), suggesting very low population frequency. According to the classification criteria of the American College of Medical Genetics and Genomics,^[6]^ the variant can be classified as “pathogenic” since it fulfils 2 strong and 1 moderate evidence: same amino acid substitution was described previously as pathogenic independently of the nucleotide sequence; de novo variant was present in a patient without a family history of the disease; and variant absent or was present at very low frequency in controls, in our case represented by the major population studies evidenced by dbSNP.

## 3 Discussion

Although dbSNP contains a high number of evenly distributed common single nucleotide variants of various types throughout *NEDD4L*, including exonic and intronic regions, only few of them were reported to have pathogenic potential (Supplementary Figure S1). In addition to the above mentioned c.2677G>A (p.Glu893Lys), 6 other PVNH7-causing *NEDD4L* missense variants were reported in the literature, namely c.623G>A (p.Arg208Gln),^[5]^ c.2035T>C (p.Tyr679His),^[3]^ c.2036A>G (p.Tyr679Cys), c.2082G>T (p.Gln694His),^[1]^ c.2177T>C (p.Leu726Pro),^[4]^ and c.2690G>A (p.Arg897Gln),^[1]^ all with regard to the most commonly used reference transcript NM_001144967 (ENST00000400345.8). From these, in the report of Broix et al all but the siblings, whose causative mutation c.2677G>A (p.Glu893Lys) had the origin in maternal mosaicism, carried the missense sequence variants c.2036A>G (p.Tyr679Cys), c.2082G>T (p.Gln694His), c.2677G>A (p.Glu893Lys), and c.2690G>A (p.Arg897Gln), respectively, in a de novo heterozygous state.^[1]^ The heterozygous variants identified in the case described by Kato et al (c.2677G>A (p.Glu893Lys) and Ma et al (c.2177T>C (p.Leu726Pro)) had a de novo origin,^[2,4]^ while the variant reported by Elbracht et al. (c.2035T>C (p.Tyr679His)) was found to originate from paternal mosaicism.^[3]^ Each of these pathogenic variants was located in the catalytic homologous to E6AP C-terminus domain of the NEDD4L protein.^[7]^ Stouffs et al reported 5 patients carrying the same variant (c.623G>A (p.Arg208Gln)), one as an isolated de novo case and the remaining 4 describing the first familial case of an affected mother and her 3 children, however, there was no information about the origin of the mother‘s allele.^[5]^ The variant identified by Stouff et al is the only known NEDD4L-associated pathogenic variant located in the WW domain responsible for protein-protein interactions.^[7]^ Despite the continuous pattern of general variability throughout the *NEDD4L* gene (Supplementary Figure S1), such aggregation of pathogenic variants in certain domains may suggest a specific mechanism of pathogenesis that could be connected to specific positions in the gene/protein. This could raise questions about the clinical effect of other loss- or gain-of-function *NEDD4L* variants residing outside these functional domains, which were, however, yet not published. An additional “likely pathogenic” PVNH7-linked variant, a translocation t (X;18)(p21.1;q21.31) having one of the breakpoints inside the C2 domain of the *NEDD4L* (between exons 5 and 6), was reported in ClinVar (ClinVarID_592171). This single submission had, however, clinical phenotype described only as “congenital malformation” and “intellectual disability” with “no assertion criteria provided” for the interpretation. Moreover, the comment to this variant mentions disruption of *PCDH10* gene on chromosome 4 further enhancing ambiguities around this variant, making this variant hardly interpretable (ClinVar accessed June 23th, 2020).

**Figure 2:**
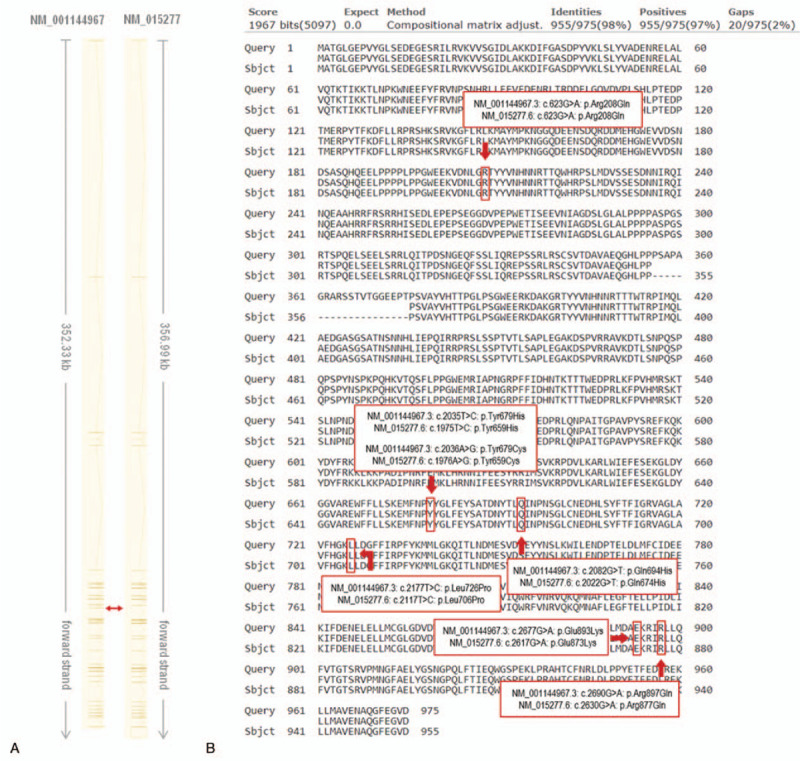
Schematic representation of differences between 2 alternative *NEDD4L* transcripts used as reference sequences by Broix et al (2016)^[1]^ and Kato et al (2017)^[2]^: (A) graphical comparison of exon/intron structure with red double arrow pointing at the site responsible for the differences in transcript length, caused by exon 13 skipping in NM_015277 when compared to NM_001144967; (B) alignment of 2 amino acid sequences translated from NM_001144967 (“subject”) and NM_015277 (“query”) generated by Protein BLAST tool. All already known/described/reported PVNH7-associated sequence variants of *NEDD4L* are marked by red arrows/frames and are denoted by human genome variation society names based on the alternative transcript. The shortening of 20 amino acids in NM_015277 transcript, likely resulting in misidentification of novel variant by Kato et al (2017),^[2]^ is clearly visible after the proline (P) at the position 355. Adapted from Ensembl.^[9]^

With regard to recurrence, the identified c.2677G>A (p.Glu893Lys) variant was reported in 5 PVNH7 patients, 4 of them by Broix et al (2016)^[1]^ and 1 by Kato et al (2017).^[2]^ It should be noted here, however, that the latter case was originally incorrectly classified by the authors as a newly discovered variant c.2617G>A (p.Glu873Lys), since the existing differences between the 2 alternative *NEDD4L* transcripts. Such misclassification may occur if the NM_015277 (ENST00000382850.8) *NEDD4L* transcript, used by Kato et al, for example, is applied as a reference for variant nomenclature, while it is shorter by 60 nucleotides than the more commonly used NM_001144967 (ENST00000400345.8) *NEDD4L* transcript (used also by Broix et al and in the present study). This may result in shifted numbering of variants, in both nucleotide and protein level, after proline at position 355 (NM_001144967.2: c.1065G; NM_015277.5: c.1065G) (Fig. 2). When reporting c.2117T>C (p.Leu706Pro), Ma et al most likely used the same NEDD4L transcript as Kato et al, although the reference used by them was not listed in the abstract of their article which was published in Chinese.^[4]^ At the given position of the variant c.2117T>C (p.Leu706Pro) we found histidine instead of leucine when using the most commonly used ENST00000400345.8 transcript. We found leucine at the 706th amino acid position (the 2117th nucleotide position) only in 2 Ensembl transcripts, ENST00000382850.8 and ENST00000256830.13. By further investigation we ruled out the ENST00000256830.13 transcript as the leucine is encoded here by TTA triplet that cannot be exchanged for proline by a single substitution. We then assumed that the ENST00000382850.8 transcript was probably used as the reference sequence by Ma et al. since the leucine is encoded here by a CTC triplet and its replacement for proline can occur by the T>C substitution. By applying these findings to the ENST00000400345.8 transcript, the originally reported position of the c.2117T>C (p.Leu706Pro) variant changes to c.2177T>C (p.Leu726Pro). These inaccuracies highlight the necessity of either listing the used transcripts in the literature or using the human genome variation society nomenclature in the sequence variants description.

When considering the present case, it is important to note that the identification of a likely causative, pathogenic *NEDD4L* variant explained just a part of our patient's phenotype, specifically those overlapping with PVNH7, such as delayed psychomotor and mental development, seizures with eyelid myoclonia and infantile spasms, periventricular nodular heterotopia, polymicrogyria, cleft palate, 2 to 3 toe syndactyly, hypotonia and hypotonic quadriparesis, microretrognathia, strabismus, and absent speech and walking. On the other hand, he presented also other distinguishable features not described among core symptoms of PVNH7, such as horizontal nystagmus and hypermetropia, thoracic scoliosis, acral hypertonia, hypsarrhythmia, gastroesophageal reflux, mild dyspnoic breathing, bilateral 5th finger clinodactyly, obstipated stool with blood admixture, suspected impairment of cholesterol metabolism and hypoproteinemia. It should be noted here that we were yet not able to confirm whether these signs are associated with his *NEDD4L* variant. It seems to be likely that symptoms shared with his older brother, that is, blue sclerae, hydronephrosis, and *talipes equinovarus,* have independent genetic etiology. Similarly to the transverse palmar crease that was found in the proband, his brother and their father as well.

## 4 Conclusion

In conclusion, our findings highlight the importance of thorough evaluation of the available literature, symptomatology and genomic findings both in probands as well as in their affected and unaffected family members, especially when reporting novel variants, or when reporting broader than previously described range of symptomatology based on single affected individuals. In addition, we advocate here a non-canonical bottom-up approach of variant filtering and prioritization which may be specifically useful in clinically complicated and ambiguous cases or families. From the view of genetic counselling, on the other, such approaches likely pose new challenges in the field of evaluation and reporting of genomic incidental findings,^[8]^ since they most probably broaden the spectrum of possibly identifiable findings during genome-wide testing.

## Author contributions

**Conceptualization:** Martina Pecimonova, Jan Radvanszky.

**Data curation:** Jaroslav Budis, Michal Lichvar.

**Formal analysis:** Martina Pecimonova, Jan Radvanszky, Jaroslav Budis.

**Funding acquisition:** Jan Turna, Tomas Szemes.

**Methodology:** Jan Radvanszky, David Smolak.

**Project administration:** Jan Turna, Tomas Szemes.

**Investigation:** Martina Pecimonova, David Smolak, Diana Kristinova, Ivica Rozova.

**Software:** Jaroslav Budis, Michal Lichvar.

**Supervision:** Jan Turna, Tomas Szemes.

**Visualization:** Martina Pecimonova, Jan Radvanszky.

**Writing – original draft:** Martina Pecimonova.

**Writing – review & editing:** Jan Radvanszky, Tomas Szemes.

## Supplementary Material

Supplemental Digital Content

## References

[1] Broix L Jagline H Ivanova E. Mutations in the HECT domain of NEDD4L lead to AKT-mTOR pathway deregulation and cause periventricular nodular heterotopia. Nat Genet 2016;48:1349–58. 27694961 10.1038/ng.3676 PMC5086093

[2] Kato K Miya F Hori I. A novel missense mutation in the HECT domain of NEDD4L identified in a girl with periventricular nodular heterotopia, polymicrogyria and cleft palate. J Hum Genet 2017;62:861–3. 28515470 10.1038/jhg.2017.53

[3] Elbracht M Kraft F Begemann M. Familial NEDD4L variant in periventricular nodular heterotopia and in a fetus with hypokinesia and flexion contractures. Mol Genet Genomic Med 2018;6:1255–60. 30393983 10.1002/mgg3.490 PMC6305664

[4] Ma J Gao J Zhang K. Clinical and genetic analysis of a patient with periventricular nodular heterotopia 7 caused by NEDD4L gene variant. Zhonghua Yi Xue Yi Chuan Xue Za Zhi 2020;37:41–3. doi:10.3760/cma.j.issn.1003-9406.2020.01.011. 31922594 10.3760/cma.j.issn.1003-9406.2020.01.011

[5] Stouffs K Verloo P Brock S. Recurrent NEDD4L variant in periventricular nodular heterotopia, polymicrogyria and syndactyly. Front Genet 2020;11:26 doi:10.3389/fgene.2020.00026. 32117442 10.3389/fgene.2020.00026 PMC7013364

[6] Richards S Aziz N Bale S. Standards and guidelines for the interpretation of sequence variants: a joint consensus recommendation of the American College of Medical Genetics and Genomics and the Association for Molecular Pathology. Genet Med 2015;17:405–24. 25741868 10.1038/gim.2015.30 PMC4544753

[7] Harvey KF Kumar S. Nedd4-like proteins: an emerging family of ubiquitin-protein ligases implicated in diverse cellular functions. Trends Cell Biol 1999;9:166–9. https://www.ncbi.nlm.nih.gov/pubmed/10322449 10322449 10.1016/s0962-8924(99)01541-x

[8] Biesecker LG. ACMG secondary findings 2.0. Genet Med 2017;19:604 doi:10.1038/gim.2017.27. 28406490 10.1038/gim.2017.27

[9] Cunningham F Achuthan P Akanni W. Ensembl 2019. Nucleic Acids Res 2019;47 (D1):D745–51. doi:10.1093/nar/gky1113. 30407521 10.1093/nar/gky1113 PMC6323964

