# Supplementary material for: Admixed phenotype of NEDD4L associated periventricular nodular heterotopia: A case report
Source: Medicine (Baltimore). 2021 Jun 4;100(22):e26136. doi: 10.1097/MD.0000000000026136 (PMC8183750; doi:10.1097/MD.0000000000026136)
Supplement: Supplemental Digital Content [file medi-100-e26136-s001.docx]

# **Supplementary information**

# **Description of the methods used for diagnostics**

Results of cytogenetic and aCGH testing were available only from the medical documentations of the proband since these tests, together with their interpretations, were performed outside of our laboratories. From the proband’s parents and his older brother, peripheral blood was collected into tubes containing EDTA for the purpose of whole exome sequencing. Genomic DNA was isolated from the whole blood using QIAamp DNA Blood Mini Kit (Qiagen). Subsequently, whole exome sequencing was performed by commercial sequencing service provided by CeGaT GmbH (Germany). This included library preparation using SureSelect Human All Exon V7 kit (Agilent) and sequencing using a NovaSeq 6000 (Illumina) platform. Processing of raw sequencing data, provided as FASTQ files from the vendor, was performed using an in house combination of different bioinformatics tools, including trimming of low quality ends of reads and adapter sequences by Trimmomatic[^1^](https://paperpile.com/c/TjVQIL/gVeH); mapping to the reference genome (hg38/GRCh38) using Bowtie2[^2^](https://paperpile.com/c/TjVQIL/jKjB); read sorting based on the mapping to genomic positions using SAMTools Sort[^3^](https://paperpile.com/c/TjVQIL/3pGm); labelling of PCR duplicates generated from the same clone using Picard Tools[^4^](https://paperpile.com/c/TjVQIL/ZioU); local realignment of sequences containing insertions and deletions by GATK[^5,6^](https://paperpile.com/c/TjVQIL/qhXS+AJEf); variant calling for identification of individual positions of variants with subsequent genotype determination in given positions was performed using Vardict[^7^](https://paperpile.com/c/TjVQIL/UO9e); and variant annotation using wANNOVAR[^8^](https://paperpile.com/c/TjVQIL/nOAs).

**References:**

1. [Bolger AM, Lohse M, Usadel B. Trimmomatic: a flexible trimmer for Illumina sequence data. *Bioinformatics*. 2014;30(15):2114-2120. doi:](http://paperpile.com/b/TjVQIL/gVeH)[10.1093/bioinformatics/btu170](http://dx.doi.org/10.1093/bioinformatics/btu170)

2. [Langmead B, Salzberg SL. Fast gapped-read alignment with Bowtie 2. *Nature Methods*. 2012;9(4):357-359. doi:](http://paperpile.com/b/TjVQIL/jKjB)[10.1038/nmeth.1923](http://dx.doi.org/10.1038/nmeth.1923)

3. [Li H, Handsaker B, Wysoker A, et al. The Sequence Alignment/Map format and SAMtools. *Bioinformatics*. 2009;25(16):2078-2079. doi:](http://paperpile.com/b/TjVQIL/3pGm)[10.1093/bioinformatics/btp352](http://dx.doi.org/10.1093/bioinformatics/btp352)

4. [Picard Tools - By Broad Institute.](http://paperpile.com/b/TjVQIL/ZioU) <http://broadinstitute.github.io/picard/.> [Accessed August 14, 2019.](http://paperpile.com/b/TjVQIL/ZioU)

5. [McKenna A, Hanna M, Banks E, et al. The Genome Analysis Toolkit: a MapReduce framework for analyzing next-generation DNA sequencing data. *Genome Res*. 2010;20(9):1297-1303.](http://paperpile.com/b/TjVQIL/qhXS)

6. [DePristo MA, Banks E, Poplin R, et al. A framework for variation discovery and genotyping using next-generation DNA sequencing data. *Nat Genet*. 2011;43(5):491-498.](http://paperpile.com/b/TjVQIL/AJEf)

7. [Lai Z, Markovets A, Ahdesmaki M, et al. VarDict: a novel and versatile variant caller for next-generation sequencing in cancer research. *Nucleic Acids Res*. 2016;44(11):e108.](http://paperpile.com/b/TjVQIL/UO9e)

8. [Chang X, Wang K. wANNOVAR: annotating genetic variants for personal genomes via the web. *J Med Genet*. 2012;49(7):433-436.](http://paperpile.com/b/TjVQIL/nOAs)

**
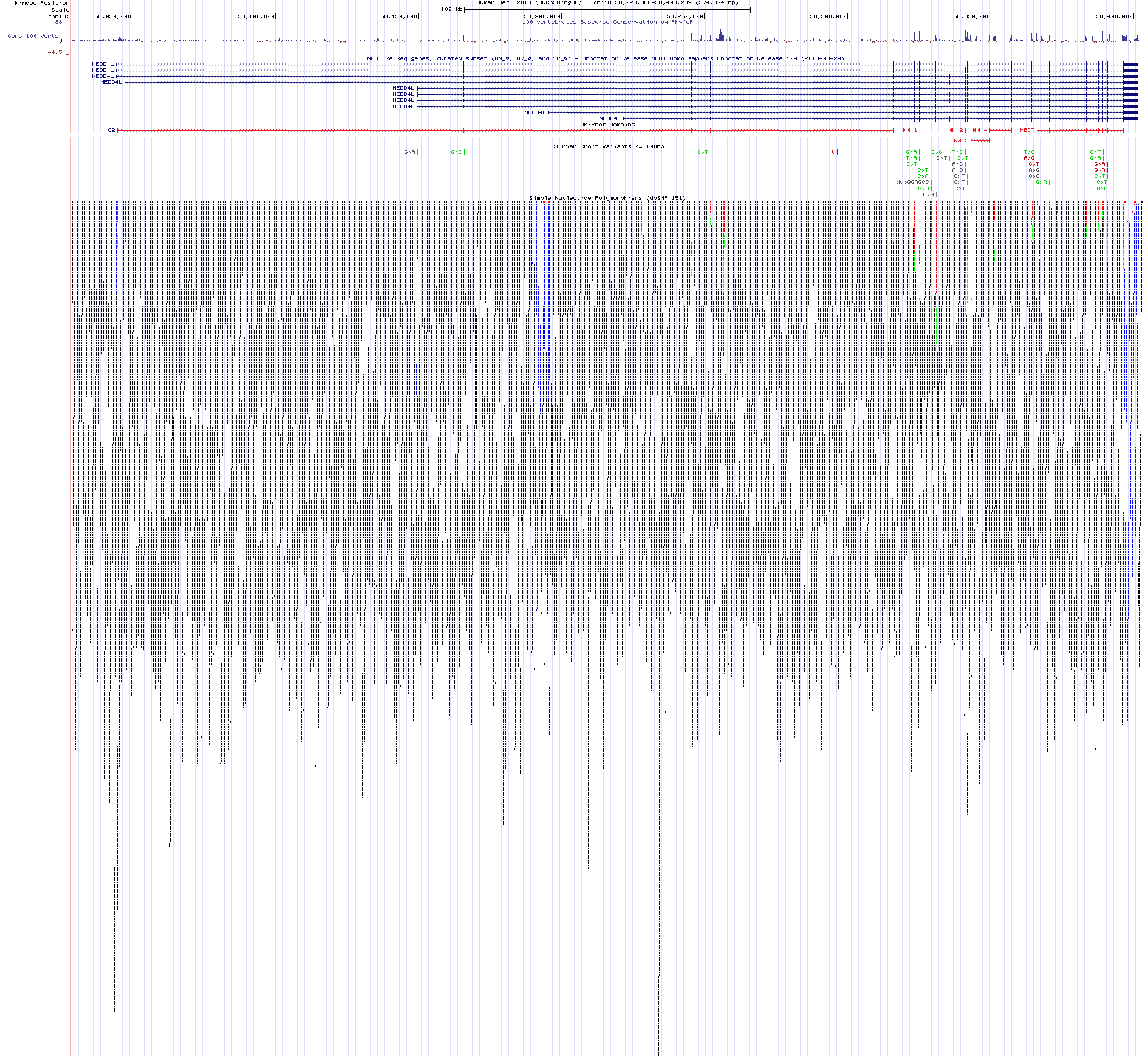
**

**Supplementary Fig. 1:** Schematic representation of the *NEDD4L* gene with conservation patterns among species, distribution of protein domains and sequence variants according to different databases. Downloaded from the UCSC Genome Browser (on Human Dec. 2013 Assembly_GRCh38/hg38) [(Haeussler et al. 2019)](https://paperpile.com/c/8RszWN/ZNIum). Tracks, according to their order from up to down are as follows: **i)** Assembly, Base Positions (chr18:58,028,866-58,403,239), Scale Bar and Ruler; **ii)** Conservation Track, 100 vertebrates Basewise Conservation by PhyloP; **iii)** NCBI RefSeq genes, curated subset (NM_*, NR_*, and YP_*), Homo sapiens Annotation Release 109 (2018-03-29); **iv)** UniProt protein domains (only those manually annotated), including an N-terminal Ca^2+^ phospholipid binding domain, 4 WW domains providing protein-protein interactions, and a C-terminal catalytic HECT (Homologous to the E6-AP Carboxyl Terminus) domain [(Harvey and Kumar 1999)](https://paperpile.com/c/8RszWN/fiMK); **v)** ClinVar Short Variants (<= 100bp) with color legend - green (benign or likely benign variants), red (pathogenic or likely pathogenic variants), grey (variants of unknown significance); **vi)** dbSNP v151 Single Nucleotide Polymorphisms with color legend - black (intron, downstream gene and upstream gene variants), green (coding synonymous variants), red (coding non-synonymous variants, including stop-gained, missense, stop-lost, in-frame indels and frameshift; as well as splice site variants, including splice-acceptor and splice-donor), blue (untranslated variants, including 5´UTR and 3´UTR; as well as non-coding (ncRNA) transcript variants).

| **Symptoms of periventricular nodular heterotopia 7 (OMIM #617201)** | **Symptoms of PVNH7 patients described by** | | | | | **Proband’s symptoms** | **Symptoms of proband’s brother** | |
| --- | --- | --- | --- | --- | --- | --- | --- | --- |
|  | **Broix et al. (2016)^1^** | **Kato et al. (2017)^2^** | **Elbracht et al. (2018)^3^** | **Ma et al. (2020)*^4^** | **Stouffs et al. (2020)^5^** |  |  |  |
| **Head and neck** | | | | | | | | |
| Dysmorphic facial features, variable (in some patients) | - | - | - | - | Prominent/high forehead (in some patients) | - | - | |
| Micrognathia | Micrognathia | - | Mild retrogenia (in a fetus of prematurely terminated pregnancy) | - | - | Microretrognathia | - | |
| Cleft palate | Cleft palate/bifid uvula | Cleft palate | Cleft palate | Cleft palate | - | Cleft palate | - | |
| Hearing impairment (in some patients) | Hearing impairment | - | - | - | - | - | - | |
| Strabismus | Strabismus, convergent/complex | - | - | - | - | Strabismus | - | |
| Optic atrophy (in some patients) | Optic atrophy | - | - | - | - | - | - | |
| - | Dysmorphic features | - | - | - | - | - | - | |
| - | Myopia | - | - | - | - | - | - | |
| - | Abnormal visual evoked potentials, optic nerve pallor | - | - | - | - | - | - | |
| - | Ptosis | - | - | - | - | - | - | |
| - | - | Unstable neck | - | - | - | - | - | |
| - | - | Difficulty maintaining eye contact | - | - | - | - | - | |
| - | - | - | Mild plagiocephaly | - | - | - | - | |
| - | - | - | Low posterior hairline | - | - | - | - | |
| - | - | - | Short neck | - | - | - | - | |
|  |  |  |  |  | Macrocephaly |  |  | |
| - | - | - | - | - | - | Blue sclerae | Blue sclerae | |
| - | - | - | - | - | - | Nystagmus, horizontal | - | |
| - | - | - | - | - | - | Hypermetropy | - | |
| - | - | - | - | - | - | - | Late dentition | |
| **Muscular system** | | | | | | | | |
| Contractures | - | - | Contractures (in a fetus of prematurely terminated pregnancy) | - | - | - | - | |
| Truncal hypotonia | Hypotonia | Hypotonia | Truncal hypotonia | Hypotonia | Hypotonia (in some patients) | Hypotonia, hypotonic quadriparesis, acral hypertonia | Mild hypotonia | |
| - | - | - | - | - | - | Hypotrophy | - | |
| **Limbs** | | | | | | | | |
| 2-3 toe syndactyly | 2-3 toe syndactyly | - | 2-3 toe syndactyly, mild | - | 2-3 toe syndactyly (in some patients) | 2-3 toe syndactyly, simple incomplete | - | |
| - | Arthrogryposis | *-* | - | - | - | - | - | |
| - | - | - | Hyperactive tendon reflexes | - | - | - | - | |
| - | - | - | Muscular hypertonia on both legs | - | - | - | - | |
| - | - | - | Adducted thumbs | - | - | - | - | |
| - | - | - | Sandal gap, bilateral | - | - | - | - | |
| - | - | - | *Talipes equinovarus*, bilateral | *-* | *-* | *Talipes equinovarus*, bilateral | *Talipes equinovarus*, bilateral | |
|  |  |  |  |  | 3-4 and 3-4-5 toe syndactyly (in some patients) |  |  | |
| - | - | - | - | - | - | Transversal crease on left hand, incomplete | Transversal crease, bilateral | |
| - | - | - | - | - | 5th finger clinodactyly | Bilateral 5th finger clinodactyly | - | |
| **Genitourinary system** | | | | | | | | |
| Cryptorchidism | Cryptorchidism | - | - | - | - | - | - | |
|  |  |  |  |  | Penoscrotal hypospadias with bifid chordee (in some patients) |  |  | |
| - | - | - | - | - | - | Hydronephrosis, grade 1 | Hydronephrosis | |
| **Nervous system** | | | | | | | | |
| Delayed psychomotor development | Developmental delay | Developmental delay | Developmental delay | Global developmental delay | - | Delayed psychomotor development | - | |
| Intellectual disability | Intellectual disability | - | - | Intellectual disability | Intellectual disability/ need of special education | Intellectual disability | - | |
| Periventricular nodular heterotopia | Periventricular nodular heterotopia | Periventricular nodular heterotopia | Periventricular nodular heterotopia | - | Periventricular nodular heterotopia | Periventricular nodular heterotopia | - | |
| Cortical dysplasia (in some patients) | Cortex anomalies (cerebral atrophy, frontal cortical dysplasia, polymicrogyria) | Polymicrogyria | Cortical malformation (polymicrogyria) | - | Perisylvian polymicrogyria (in some patients) | Polymicrogyria | - | |
| Thin *corpus callosum* (in some patients) | Dysmorphic *corpus callosum* | - | - | - | - | - | - | |
| Seizures (in some patients) | Seizures, infantile spasms | Infantile spasm | - | Seizures | Seizures/convulsion (in some patients) | Seizures with eyelid myoclonia, infantile spasms | - | |
| Poor or absent speech | - | - | - | - | Absent speech | Absent speech | - | |
| Delayed or absent walk | Absent walk | - | - | - | - | Absent walk | - | |
| - | - | - | Mild thickening of the *cavum septi pellucidi* (in a fetus of prematurely terminated pregnancy) | - | - | - | - | |
|  |  |  |  |  | - |  |  | |
| - | - | - | - | - | - | Decreased pain sensitivity | Decreased pain sensitivity | |
| **Circulatory system** | | | | | | | | |
| - | - | Hypsarrhythmia | - | - | - | Hypsarrhythmia | - | |
| - | - | Patent foramen ovale | - | - | - | - | - | |
| **Metabolism and digestive system** | | | | | | | | |
| - | - | - | - | - | - | Gastroesophageal reflux | - | |
| - | - | - | - | - | - | Obstipated stool often with admixture of blood | - | |
| - | - | - | - | - | - | Suspected impairment of cholesterol metabolism (mildly decreased levels of total and HDL cholesterol) | - | |
| - | - | - | - | - | - | Hypoproteinemia (mildly decreased levels of A and B apolipoproteins) | - | |
| **Other** | | | | | | | | |
| - | - | - | Hypokinesia (in a fetus of prematurely terminated pregnancy) | - | - | - | - | |
| - | - | - | - | - | - | Growth retardation | - | |
| - | - | - | - | - | - | Subclinical hypothyreosis | - | |
| - | - | - | - | - | - | Thoracic left convex scoliosis | - | |
| - | - | - | - | - | - | Mild dyspneic breathing | - | |

**Supplementary Table 1:** Summarization of all known symptoms associated with periventricular nodular heterotopia-7 (PVNH7) listed in OMIM® (Online Mendelian Inheritance in Man) database and reported by Broix et al. (2016)[^1^](https://paperpile.com/c/7pS7Xk/6z8A), Kato et al. (2017)[^2^](https://paperpile.com/c/7pS7Xk/kBQ7), Elbracht et al. (2018)[^3^](https://paperpile.com/c/7pS7Xk/zUAI), Ma et al. (2020)^4^ and Stouffs et al. (2020)^5^ and their comparison with complete phenotypic manifestation of the patient/proband and his older brother. “-“ absent or unexamined feature. „*“ as the full article is in Chinese, features listed in this table include those reported in the English abstract, therefore it is not excluded that the patient presented with more clinical features.

**References**

1. [Broix L, Jagline H, Ivanova E, et al. Mutations in the HECT domain of NEDD4L lead to AKT-mTOR pathway deregulation and cause periventricular nodular heterotopia. *Nat Genet*. 2016;48(11):1349-1358. doi:](http://paperpile.com/b/bEsFxP/pNlAe)[10.1038/ng.3676](http://dx.doi.org/10.1038/ng.3676)

2. [Kato K, Miya F, Hori I, et al. A novel missense mutation in the HECT domain of NEDD4L identified in a girl with periventricular nodular heterotopia, polymicrogyria and cleft palate.](http://paperpile.com/b/bEsFxP/NyeX) *[J Hum Genet](http://paperpile.com/b/bEsFxP/NyeX)*[. 2017;62(9):861-863. doi:](http://paperpile.com/b/bEsFxP/NyeX)[10.1038/jhg.2017.53](http://dx.doi.org/10.1038/jhg.2017.53)

3. [Elbracht M, Kraft F, Begemann M, et al. Familial NEDD4L variant in periventricular nodular heterotopia and in a fetus with hypokinesia and flexion contractures. *Mol Genet Genomic Med*. 2018;6(6):1255-1260. doi:](http://paperpile.com/b/bEsFxP/nwJnh)[10.1002/mgg3.490](http://dx.doi.org/10.1002/mgg3.490)

4. [Ma J, Gao J, Zhang K, et al. [Clinical and genetic analysis of a patient with periventricular nodular heterotopia 7 caused by NEDD4L gene variant]. *Zhonghua Yi Xue Yi Chuan Xue Za Zhi*. 2020;37(1):41-43. doi:](http://paperpile.com/b/bEsFxP/YF6No)[10.3760/cma.j.issn.1003-9406.2020.01.011](http://dx.doi.org/10.3760/cma.j.issn.1003-9406.2020.01.011)

5. [Stouffs K, Verloo P, Brock S, et al. Recurrent NEDD4L Variant in Periventricular Nodular Heterotopia, Polymicrogyria and Syndactyly. *Front Genet*. 2020;11:26. doi:](http://paperpile.com/b/bEsFxP/YDF3T)[10.3389/fgene.2020.00026](http://dx.doi.org/10.3389/fgene.2020.00026)
